# Changes in the Expression of miR-381 and miR-495 Are Inversely Associated with the Expression of the MDR1 Gene and Development of Multi-Drug Resistance

**DOI:** 10.1371/journal.pone.0082062

**Published:** 2013-11-26

**Authors:** Yan Xu, Stephen J. Ohms, Zhen Li, Qiao Wang, Guangming Gong, Yiqiao Hu, Zhiyong Mao, M. Frances Shannon, Jun Y. Fan

**Affiliations:** 1 School of Life Sciences and Technology, Tongji University, Shanghai, People’s Republic of China; 2 Department of Genome Biology, John Curtin School of Medical Research, The Australian National University, Canberra, Australia; 3 State Key Laboratory of Pharmaceutical Biotechnology, Nanjing University, Nanjing, People’s Republic of China; 4 Australian Cancer Research Foundation Biomolecular Resource Facility, John Curtin School of Medical Research, The Australian National University, Canberra, Australia; 5 National Information and Communications Technology Australia, Victoria Research Laboratory, The University of Melbourne, Melbourne, Australia; 6 The University of Canberra, Canberra, Australia; National Institutes of Health, United States of America

## Abstract

Multidrug resistance (MDR) frequently develops in cancer patients exposed to chemotherapeutic agents and is usually brought about by over-expression of P-glycoprotein (P-gp) which acts as a drug efflux pump to reduce the intracellular concentration of the drug(s). Thus, inhibiting P-gp expression might assist in overcoming MDR in cancer chemotherapy. MiRNAome profiling using next-generation sequencing identified differentially expressed microRNAs (miRs) between parental K562 cells and MDR K562 cells (K562/ADM) induced by adriamycin treatment. Two miRs, miR-381 and miR-495, that were strongly down-regulated in K562/ADM cells, are validated to target the 3’-UTR of the *MDR1* gene. These miRs are located within a miR cluster located at chromosome region 14q32.31, and all miRs in this cluster appear to be down-regulated in K562/ADM cells. Functional analysis indicated that restoring expression of miR-381 or miR-495 in K562/ADM cells was correlated with reduced expression of the MDR1 gene and its protein product, P-gp, and increased drug uptake by the cells. Thus, we have demonstrated that changing the levels of certain miR species modulates the MDR phenotype in leukemia cells, and propose further exploration of the use of miR-based therapies to overcome MDR.

## Introduction

Multidrug resistance (MDR) is one of the main obstacles to the successful treatment of cancer patients with chemotherapeutic agents. As a prevalent clinical phenotype, cancer cells from patients who have been exposed to one chemotherapeutic agent, become resistant to that agent and consequently develop cross-resistance to a wide range of other chemotherapeutic agents [1]. Efflux of hydrophobic drugs out of cells is the most commonly encountered mechanism of MDR. ATP-binding cassette (ABC) transporters, a superfamily of transmembrane proteins, play pivotal roles in this process [2]. Among them, P-glycoprotein (P-gp), encoded by the *MDR1* gene (also known as the *ABCB1* gene), is the most well studied [3,4].

*MDR1*/P-gp is expressed naturally in important physiological barriers such as the gastrointestinal tract, liver and kidney, where it acts as a barrier for protection against endogenous and exogenous toxins [5]. In addition to its physiologic expression in normal tissues, it is also expressed and, mostly, over-expressed, in certain human tumors [6]. Tumors derived from tissues expressing P-gp are normally resistant to chemotherapy. In contrast, some other tumors, such as breast tumors and leukemia, with low or no expression of P-gp, develop MDR only after treatment with anti-cancer drugs when the over-expression of P-gp is induced [7].

Therapeutic use of P-gp inhibitors to overcome MDR was first proposed in 1981 when Tsuruo et al. found that verapamil could reverse MDR in Vincristine-resistant leukemia cells P388/VCR [8]. To date, three generations of P-gp inhibitors have been developed, mostly competitive or non-competitive inhibitors targeting P-gp with increasing potency and decreasing toxicity [9]. However, none of them have been clinically successful because of their unwanted pharmacokinetic interactions with chemotherapeutic agents or because of a lack of specificity [10]. Clearly, modulation of P-gp expression could also potentially reverse the phenotype of MDR and it has been previously shown that P-gp activity against certain substrates can be significantly modulated by specific mutations. A synonymous SNP in the *MDR1* gene, C3435T, which does not cause an amino acid substitution, was reported to be associated with low intestinal P-gp expression, low P-gp activity, and high digoxin absorption in individuals carrying this allele [11]. In addition, a T3587G germ-line mutation of *MDR1* expresses a non-functional P-gp [12]. However, little is known about the mechanisms regulating expression of the *MDR1* gene. A study, using the human leukemia K562 cell line and its multidrug-resistant derivative, K562/ADM, revealed that DNA demethylation at the repressor binding site (the -110 GC-box) of the *MDR1* gene in K562/ADM cells is associated with up-regulation of P-gp expression [13]. Recent studies suggest that *MDR1* is also regulated by different miRs in different tumour types. For example, miR-27a and miR-451 are activators of *MDR1*/P-gp expression in the development of MDR in human ovarian and cervical cancer cells [14] while miR-451 negatively regulates the expression of the *MDR1* gene in the multidrug-resistant breast cancer cell line, MCF7/DOX [15]. The growing evidence of regulation of P-gp expression by miRs led us to investigate the possibility of using a miR-based approach to silence P-gp over-expression in human multidrug-resistant leukemia cells.

Several technologies, such as microarrays and PCR-based arrays, have been developed for genome-wide miR expression profiling [16]. However, massively parallel sequencing, which not only provides accurate measurements of miR profiles but also enables the identification of novel miRs and other small RNAs, has not been widely utilized in miR screening. Taking advantage of this technology, we investigated the differentially-expressed miRs in the K562 human leukemia cell line, (derived from a chronic myelogenous leukemia patient) and multidrug-resistant K562/ADM cells, and identified and validated key miR candidates whose expression is inversely related to that of P-gp. We also present evidence that modulation of miR expression reduces the effects of the MDR phenotype with drug uptake being increased in MDR leukemia cells treated with adriamycin or vinblastine.

## Materials and Methods

### Cell culture and generation of MDR cell lines

Human chronic myelogenous leukemia K562 cells bought from ECACC (Sigma) were cultured in complete RPMI 1640 medium with 10% fetal bovine serum (FBS) and 1% penicillin/streptomycin at 37°C in a humidified atmosphere containing 5% CO_2_.

A stable adriamycin (ADM, Sigma)-resistant cell line variant (K562/ADM) was established from K562 by continuous exposure of the cells to increasing concentrations of ADM up to 1000 ng/mL. Subsequently, K562/ADM cells were cultured in the presence of 1000 ng/mL of ADM to maintain the drug-resistant phenotype. Similarly, another multidrug-resistant cell line, K562/VBL, was established from K562 by continuous exposure of the cells to an increasing concentration of vinblastin (VBL, Sigma) up to 500 ng/mL.

HCA2-hTERT cells were cultured in MEM media with 15% fetal bovine serum, 100 units/mL penicillin and 100μg/mL streptomycin at 37°C.

### cDNA synthesis and real time-PCR

Total RNA was prepared from cells using TriReagent (Sigma-Aldrich) according to the manufacturer’s instructions. RNA (1µg) was treated with DNase I (1U) in Tris buffer (pH 7.6) containing 5 mM MgCl_2_ at 37°C for 30 min, and reverse-transcribed using first-strand cDNA Synthesis (Marligen, USA) as detailed in the manufacturer’s instructions. Real-time PCR reactions were performed with 50 ng of cDNA and Power SYBR® Green PCR Master Mix (ABI) in a total volume of 20 µL on an ABI 7500 sequence detector (Applied Biosystems). Details of the primers used for the measurement of gene expression are listed in Table S1. The Ct values for the genes of interest were normalized to that of Glyceraldehyde 3-phosphate dehydrogenase (GAPDH) whose expression did not change in response to the drug treatment.

### Surface staining and FACS analysis

To analyze the expression of P-gp, K562, K562/ADM and K562/VBL cells (approximately 1×10^6^ cells) were stained with 20 µL monoclonal anti-human P-gp 17F9 antibody labeled with PE (BD Biosciences) for 30 min at 4°C in the dark. The binding affinity of the P-gp antibody was measured by a FACSCalibur flow cytometer (Becton Dickinson, USA). The data were analyzed using FlowJo software.

### MTT assay

Increasing concentrations of VBL dissolved in 50 μL RPMI-1640 were added to cell suspensions (1×10^5^/mL, 200 μL) in 96-well culture plates and incubated for 48 h. RPMI-1640 (50 μL) without VBL was added to the cells as a negative control and RPMI-1640 (250 μL) without cells was used as a blank control. MTT (20 μL of 5 mg/mL) was added and incubated for an additional 4 h.

### FACS analysis of Rhodamine 123 accumulation

Rhodamine 123 (Rh123, Sigma) is a fluorescent substrate of P-gp that has been extensively used as an index of P-gp-mediated transport in MDR cells[17]. Both K562 and K562/ADM cells were collected and incubated with 5 µM Rh123 at 37°C for 30 min. Cells were washed 3 times and resuspended in chilled PBS prior to measurement of intracellular Rh123 content with a FACSCalibur flow cytometer.

### Next-generation sequencing and bioinformatic analysis of the miRNAome

Total RNA was isolated from K562 and K562/ADM cells and enriched using PEG to precipitate HMW RNAs[18]. Small RNA libraries for sequencing were prepared based on Illumina’s alternative v1.5 protocol and a published method [18], and sequenced on an Illumina Genome Analyzer IIx at the ACRF Biomolecular Resource Facility of the Australian National University.

Mapping of Illumina short-read data was carried out with the miRanalyzer web server [19]. For both the K562 and K562/ADM small RNA-Seq datasets, files containing the 36-mer reads in fastq format were first processed using a Perl script (groupGAreads.pl) downloaded from the miRanalyzer website. This script condenses each fastq file into a smaller file containing each unique read and the number of times it occurs and greatly reduces the size of the files to be uploaded. Singleton reads were omitted from further analysis. The condensed read files were uploaded to the miRanalyzer web server where the Illumina adapter sequence was identified and removed from each read before mapping with the Bowtie alignment program against the “miRBase 16” database and other libraries of transcribed sequences. Default parameters for miRanalyzer were used throughout. Following mapping, normalization of the reads and analysis of differential expression was carried out with the R/Bioconductor DESeq package built-in to miRanalyzer[20].

### Validation of miRs

Expression profiling of mature miRs was determined using stem-loop real-time PCR with minor modifications [21,22]. Specific stem-loop real-time primers uniquely recognizing each miR were designed according to Chen et al. [22] (Table S2). Following initial denaturing, annealing and reverse transcription using Superscript III reverse transcriptase (Invitrogen), products underwent real-time PCR using Power SYBR® Green PCR Master Mix. Their Ct values were normalized to that of miR-425 whose expression did not change in response to the drug treatment.

### EGFP Reporter Assay for targeting the 3'-UTR of MDR1

 The 3’-untranslated region (UTR) segment of the MDR1 gene corresponding to a region of 611 bp (nucleotide positions 4,262-4,872 of the total transcript) (accession no. NM_000927.3) was amplified by PCR from human cDNA using primers with Not I tails on both forward and reverse strands. PCR products were digested with Not I restriction endonuclease and then ligated into a pEGFP-N1 vector (Clontech) at the Not I site located upstream of the polyadenylation signals of enhanced green fluorescent protein (EGFP). The EGFP-MDR1 3’-UTR reporter vector construct (25 ng) was co-transfected with 100 nM of the miR-381 or miR-495 mimics or negative control miRs into HCA2-hTERT cells using a Nucleofector 4D (Lonza). pDsRed2-N1 vector (5 ng) was co-transfected as an internal control. Two days after transfection, cells were collected and analysed on a FACSCalibur flow cytometer [23].

### MiR Transfection

Custom designed miR mimics for miR-381, miR-495 and a negative control with a scrambled sequence were synthesized by Sigma (Table S3). Cell transfections were performed with Nucleofector kits on a Nucleofector I or Nucleofector 4D. Transfections were optimized according to the manufacturer’s protocol. Briefly, 1×10^6^ cells were resuspended in Nucleofector solution containing 100 nM of miR-381, miR-495, mimics or scrambled sequences as a negative control miR. The cell mixtures were transfected in Nucleovettes in the Nucleofector machine with the appropriate program. Similarly, miRCURY LNA^TM^ miR inhibitors (EXIQON) (Table S4) for miR-381, miR-495 and the scrambled sequence negative control were delivered into K562/ADM cells that had been pre-transfected with mimics. 

### Microscopy images of intracellular Rh123

MiR-transfected cells were incubated with 5 µM Rh123 at 37°C for 30 min [24]. Cells were washed 3 times with ice cold PBS before microscopic images were collected with an Olympus IX71 inverted microscope (Melville, NY) with DP Controller and DP Manager Software.

### Statistical analysis

The results are expressed as mean ± standard deviation (SD) of at least three independent experiments. The statistical significance was calculated with a student’s t-test and differences were considered statistically significant at P < 0.05.

## Results

### Generation of multidrug-resistant leukemia cell line

**Figure 1:**
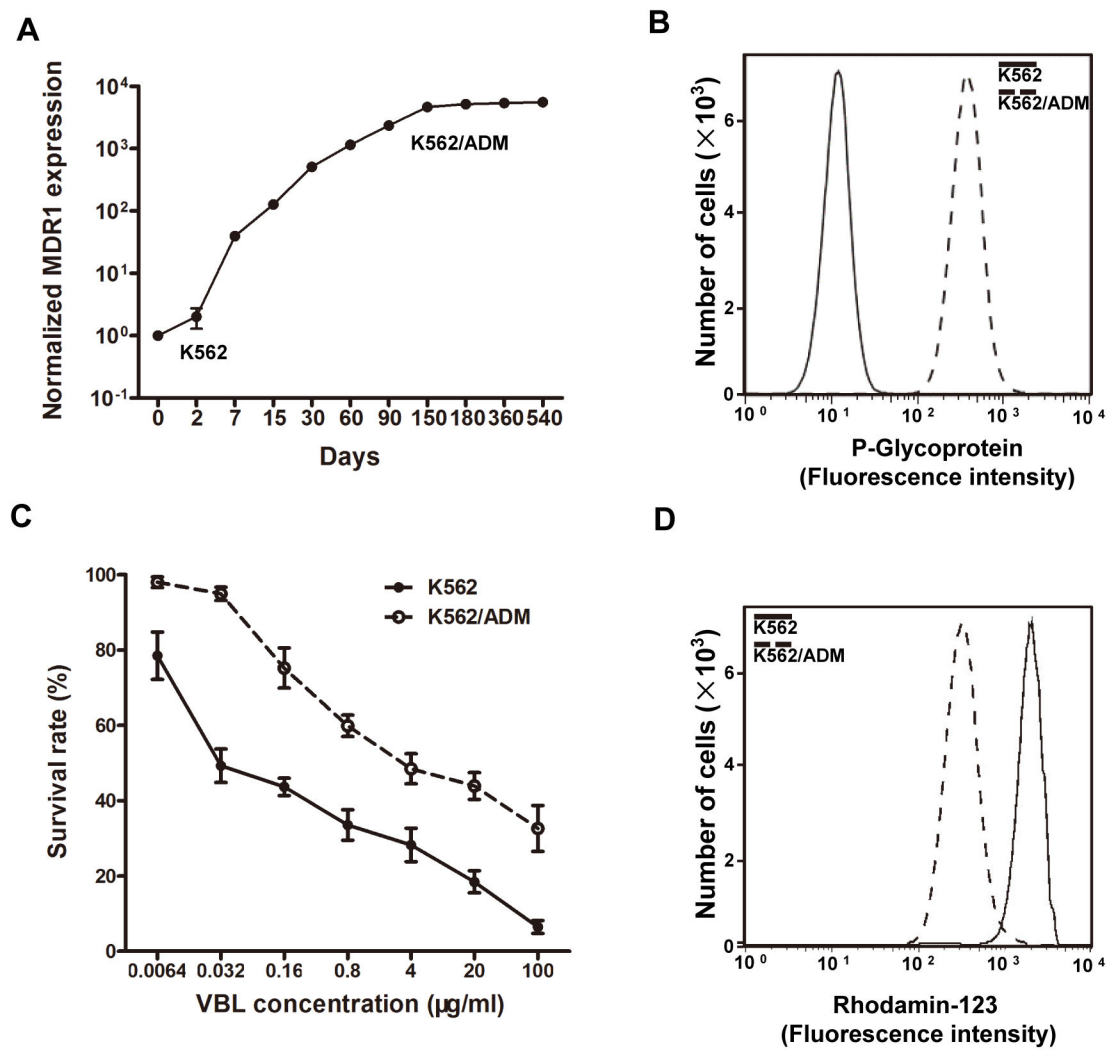
Generation of K562/ADM cells. (A) K562/ADM cells were established after exposure of K562 cells to ADM. MDR1 expression in parental and drug-treated K562 cells was normalized to the GAPDH housekeeping gene. (B) FACS analysis of K562 and K562/ADM cells stained with P-gp antibody. (C) MTT assay shows survival rate of K562 and K562/ADM cells under VBL treatment. The ratio of OD_490_ of VBL-treated cells *versus* control cells was used as the measure of survival rate. At least three independent experiments were performed and error bars represent standard deviation (S.D.). (D) FACS analysis of Rh123 accumulation in K562 and K562/ADM cells.

To analyze the changes in miR profiles during the development of MDR in leukemia cells, we first generated a MDR cell line from drug-sensitive K562 cells. K562 cells were treated with ADM for a prolonged period of time (approximately 5 months), leading to morphological changes and the development of a stable cell line referred to as K562/ADM (Figure S1A). To confirm that K562/ADM had acquired the MDR phenotype, we first examined the expression of P-gp at both the mRNA and protein levels. An approximate 5000-fold increase in *MDR1* mRNA expression was stably achieved in K562/ADM cells compared to the parental K562 cells after prolonged induction with ADM (Figure 1A). The levels of P-gp in both K562 and K562/ADM cells were determined by cell surface staining with an anti-P-gp antibody followed by FACS analysis (Figure 1B). In contrast to the parental K562 cells with low P-gp expression, a single population of K562/ADM cells with elevated P-gp expression was produced. The substrate efflux function of P-gp was verified by measuring the cell survival rate during exposure to the anticancer drug VBL in MTT assays, and by measuring the accumulation of Rh123 in cells by FACS analysis (Figure 1C and 1D). Both VBL and Rh123 are substrates of P-gp. MTT assays revealed that a higher concentration of VBL was needed to inhibit cell growth in K562/ADM cells than in K562 cells and Rh123 accumulation assays indicated that far less substrate accumulated in K562/ADM cells probably due to P-gp over-expression in this cell line compared to the parental cells, suggesting that K562/ADM is an MDR cell line.

Another MDR cell line (K562/VBL) induced by VBL exposure developed a phenotype similar to K562/ADM with elevated expression of *MDR1*/P-gp (Figure S1). 

### Genome-wide miR expression profiling in K562 and K562/ADM cells

Small RNA libraries were prepared from the K562 and K562/ADM cells as described in Materials and Methods and sequenced on an Illumina Genome Analyzer IIx. Mapping of the Illumina reads was carried out on the miRanalyzer web server [19]. For both the K562 and K562/ADM samples, about 30 million raw reads were obtained, from which 905,747 and 644,790 non-singleton unique reads respectively, were extracted along with their copy numbers. A brief summary of the reads mapping to known mature miR sequences and other transcribed libraries is presented in Table 1. After mapping and aligning these candidates to the 1032 recorded sequences of mature miRs in “miRBase 16”, 564 and 587 mature miRs were found to be expressed in K562 and K562/ADM cells, respectively.

**Table 1:** Mapping results for small RNA-Seq data.

**Mapping results**	**Samples**	
	**K562**	**K562/ADM**
Total number of reads	27,387,744	31,350,659
Total number of unique reads excluding singleton reads	905,747	644,790
Unique reads mapped to other transcribed libraries (RefSeq_genes and Rfam)	74,495	72,903
Unique reads of mature miRs	32,822	33,120
Number of known mature miRs recorded in miRBase16	1,032	1,032
Number of different types of mature miRs detected in sample	564	587

**Figure 2:**
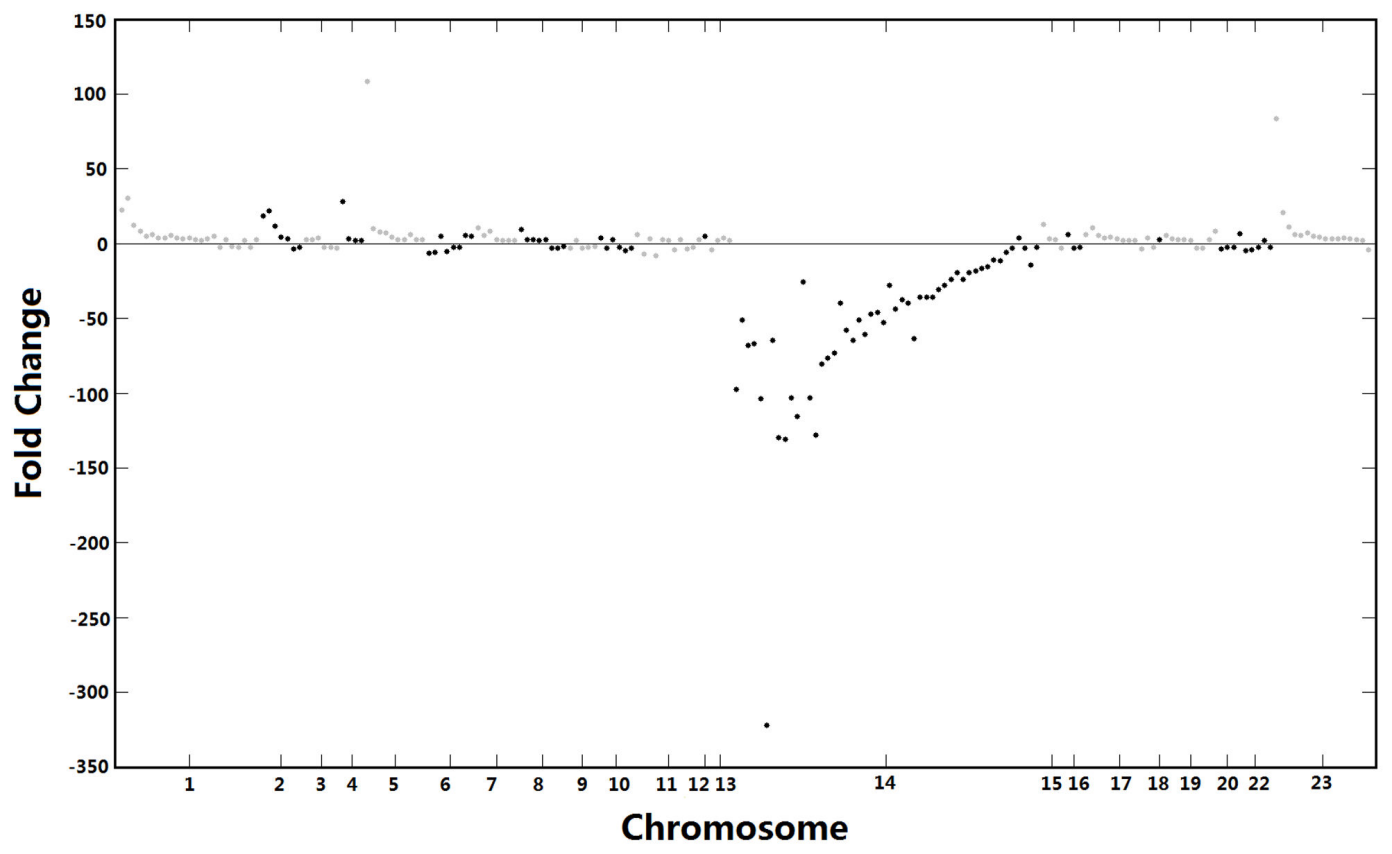
Manhattan plot of the chromosomal distribution of differentially expressed miRs. The x-axis represents the 23 human chromosomes end-to-end; the y axis shows the expression changes of individual miRs in K562/ADM cells based on miR-seq profiling. Only differentially-regulated miRs showing 2-fold or greater changes in their expression are shown. Grey dots represent miRs on chromosomes 1, 3, 5, 7, and 9 etc. while black dots are miRs on even numbered chromosomes. No miR with a 2-fold or greater fold change mapped to chromosome 21.

The raw counts of reads mapping to known miRs were then input to the DESeq package as implemented in miRanalyzer for normalization of the two libraries and calculation of fold changes of miRs between the two samples. This revealed numerous large differences in the miR profiles of the two cell types. Of the differentially expressed miRs with raw p-values for differential expression less than 0.01, most were down-regulated in K562/ADM cells. The differentially expressed miRs with fold changes greater than 2 were selected and mapped to the UCSC human genome assembly (GRCh37/hg19, February 2009) using the BWA short read aligner. Figure 2 represents the fold changes of these selected miRs in MDR cells mapped across all of the human chromosomes. Notably, a large proportion of miRs that were down-regulated in MDR cells mapped to locations on chromosome 14.

Figure 2 also presents an overview of the chromosomal distribution of up-regulated miRs with fold change > 2. In addition, up-regulated miRs with fold-changes > 10 are listed in Table S5. Interestingly, there are not many miRs displaying >10-fold increase in expression in K562/ADM cells. This leads us to a further speculation that the dysregulated miRs are those that are down-regulated in MDR cells. 

### Identification and validation of candidate miRs

**Figure 3:**
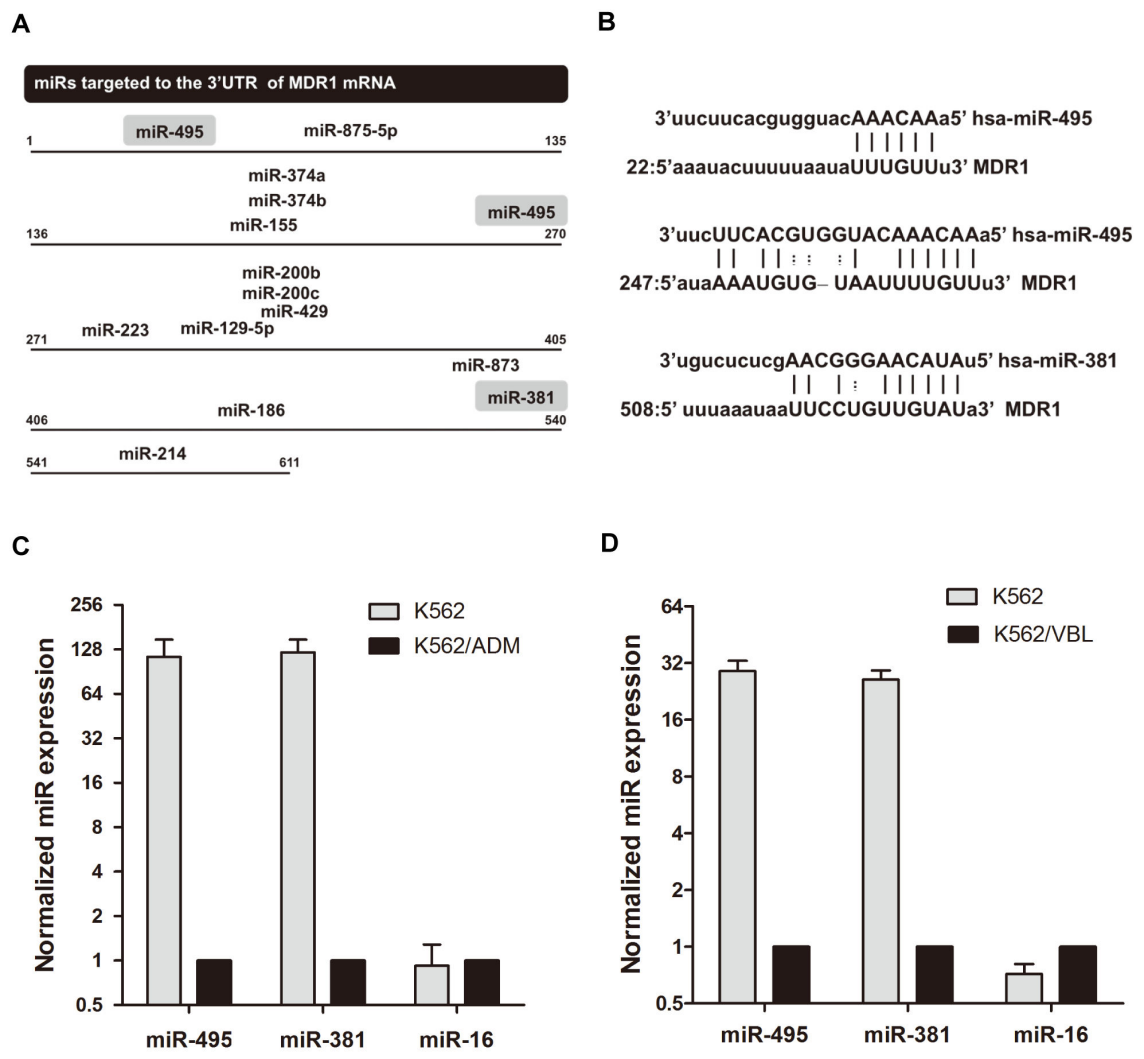
Validation of mature miR expression. (A) Schematic representation of miRs targeted to the 3'-UTR of the MDR1 mRNA predicted by microRNA.org. MiR-381 and miR-495 are highlighted and their predicted seed binding regions are shown in (B). Numbers at both ends of the fragments represent the relative nucleotide locations in this region. (C) Expression of mature miR-381, miR-495 and miR-16 in K562 and K562/ADM cells was validated by stem-loop real-time PCR. The Ct values of these miRs were normalized to that of miR-425 whose expression was unchanged in both cell types. Next, the relative expression of miR-381, miR-495 and miR-16 in K562 cells and in K562/ADM cells was calculated. The results are shown as the mean ± SD of three independent experiments. (D) The Ct values of miR-381, miR-495 and miR-16 in K562 and K562/VBL cells were normalized to miR-425. The relative expression of miR-381, miR-495 and miR-16 in K562 cells and in K562/VBL cells was calculated. The results are shown as the mean ± SD of three independent experiments.

Using the microRNA.org and TargetScan databases, approximately 14 of the down-regulated miRs are predicted to target the 3’-UTR of the *MDR1* gene (Figure 3A and 3B). Three of them, miR-381, miR-485-3p and miR-495, were down-regulated approximately 100-fold in K562/ADM cells compared to K562 cells (Figure 2 and Table 2). However, miR-485-3p is poorly conserved in the sequence region that putatively binds to the MDR1 3'-UTR according to TargetscanHuman 6.2. Thus, the other two miRs, miR-381 and miR-495, were selected as candidates for further study.

**Table 2:** miRs with >50-fold reduction in expression in K562/ADM cells and their corresponding target genes predicted by microRNA.org or TargetscanHuman 6.2.

**miR-ID**	**Fold change of reduction in expression**	**Predicted Targets**	
		**microRNA.org**	**TargetscanHuman 6.2**
miR-381	98	MDR1,ABCC5	
miR-127-3p	51		
miR-411	68	ABCG2,	
miR-379	67	ABCC1,ABCC2	ABCC2
miR-299-3p	104		
miR-494	322	SRI,GLO-1	GLO-1,ABCC5
miR-376c	65	SRI	ABCC4
miR-376a	130		
miR-369-3p	131		GLO-1,ABCC5
miR-380	103		GLO-1
miR-409-3p	116		
miR-485-3p	103		MDR1
miR-487b	128		
miR-495	80	MDR1,SRI,ABCC4,ABCC5	MDR1
miR-299-5p	76		ABCC1,ABCC4
miR-410	73	ABCC1,ABCC5	ABCG2
miR-376b	58		
miR-377	65		
miR-889	51		ABCC5
miR-485-5p	60		ABCC1
miR-409-5p	53		ABCG2
miR-323-3p	64		ABCG2

All the miRs listed below are located at chromosome region 14q32.31.

The expression of miR-381 and miR-495 was validated by the stem-loop real-time PCR method. MiR-16 was selected as a negative control and miR-425 as an internal control for Ct value calculations because miR-425 showed very little difference between K562 and K562/ADM cells from the genome-wide expression profiling data. Compared to parental K562 cells, there was an approximately 100-fold decrease in expression, by stem-loop real-time PCR, of miR-381 and miR-495 in K562/ADM cells (Figure 3C), supporting the results from the genome-wide study (Figure 2 and Table 2).

Similar down-regulation of miR-381 and miR-495 was also observed in the other MDR cell line, K562/VBL, which also expressed *MDR1*/P-gp at high levels similar to K562/ADM (Figure 3D; Figure S1), suggesting a common phenomenon in MDR leukemia cells in which *MDR1* gene expression is negatively correlated with miR-381 and/or miR-495 expression levels.

### MiR-381 and miR-495 expression is negatively correlated with MDR1/ P-gp expression and function

**Figure 4:**
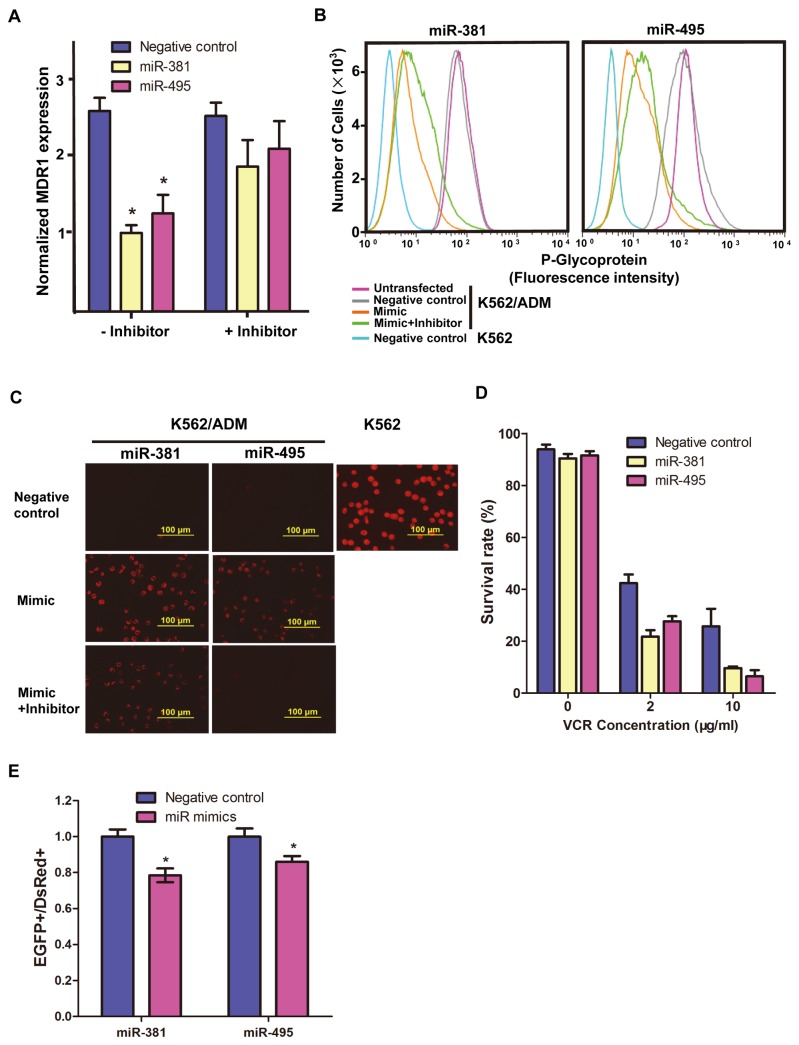
Functional analysis of miR-381 and miR-495 in K562/ADM cells. (A) After transfection of K562/ADM cells with mimics of miR-381, miR-495 or negative controls, or transfection with inhibitors in addition to mimics, MDR1 mRNA expression was determined by real-time PCR. Expression values were normalized to GAPDH and are shown as the mean ± SD of three independent experiments. * indicates a significant difference between the two groups of samples with p<0.05 calculated using a two-sample t-test. (B) FACS analysis showed the different levels of P-gp expression in K562 cells and K562/ADM cells without transfection, or transfected with scrambled sequences as negative controls or mimics alone or mimics plus inhibitors of miR-381 or miR-495. (C) Microscopy images of Rh123 accumulation in K562 and K562/ADM cells with different treatments. Each sample had a cell density of approximately 1×10^6^ cells/mL. (D) Cell survival rate after transfection with mimics of miRs in the presence of 0, 2 or 10 µg/mL VCR. Data is shown as the mean ± SD of three independent experiments. (E) Relative GFP fluorescence intensity compared to internal control DsRed was analyzed by FACS after co-transfection with mimics of miR-381 or miR-495 or negative control. * indicates a significant difference between the two groups of samples with p<0.05 calculated using a two-sample t-test.

The functional importance of miR-381 and miR-495 in relation to *MDR1*/P-gp expression was evaluated by manipulating their levels using mimics and inhibitors of these miRs, with scrambled sequences as negative controls. As shown in Figure 4A, K562/ADM cells transfected with miR-381 and miR-495 mimics showed a 63% and 54% reduction in *MDR1* expression, respectively. In contrast, transfection with each inhibitor in the mimic-treated cells led to increased *MDR1* expression with no changes being observed in the negative control. Changes in P-gp protein expression in the transfected K562/ADM cells were confirmed by FACS analysis, and were consistent with the changes observed in mRNA expression (Figure 4B).

We experienced difficulties in performing experiments with either the miR-381 or miR-495 inhibitors transfected into parental K562 cells. No obvious change in P-gp expression occurred when low concentrations of the inhibitor (100 nM) were used to transfect K562 cells. However, further increases in the concentrations of the inhibitors caused serious toxicity eventually leading to cell death (data not shown).

To further confirm the potential role of miR-381 or miR-495 in regulating P-gp expression, we assessed the membrane transporter activity of P-gp by observing intracellular Rh123 content using fluorescence inverted microscopy (Figure 4C). Rh123 accumulation was increased in K562/ADM cells treated with mimics of miR-381 or miR-495, but this accumulation was counteracted upon transfection with the inhibitors, indicating that the miRs could reverse the *MDR1*/P-gp expression and thus restore substrate accumulation.

Cytotoxicity of K562/ADM cells under treatment with Vincristine (VCR) was analyzed by counting the number of live cells using Muse™ Count and Viability kits. As shown in Figure 4D, in comparison to the negative control group, miR-495 or miR-381-transfected cells were more prone to cell death. In the treatment with 2 µg/mL VCR, the survival rate of miR transfected cells was approximately half that of control cells. A further increase of VCR concentration to 10 µg/mL led to a 60% and 75% reduction of survival rate in both miR-transfected cells compared with non-transfected cells, suggesting that the two miRs can potentially improve the efficiency of chemotherapy.

To further confirm that the changes in expression of miR-381 and miR-495 were associated with changes in expression of the MDR1 gene and the development of multi-drug resistance, we constructed a pEGFP-MDR1 3’UTR vector containing the 3’-untranslated region (UTR) segment of *MDR1*. By transiently introducing miR-381 or miR-495 mimics together with the pEGFP-MDR1 3’UTR and an internal control (pDsRed2-N1) for normalizing transfection efficiency into HCA2-hTERT, a human foreskin fibroblast cell line, we found that GFP expression decreased by approximately 20% in the presence of miR-381 or miR-495 (Figure 4E), proving that the two miRs can modulate expression of the MDR1 gene by targeting its 3'-UTR.

UTR reporter assays with other miRs, miR-376a and miR-369-3p, that have strongly reduced expression in K562/AMD cells but are not predicted to target the MDR1 gene were also performed. No obvious change in GFP expression was observed (Figure S2), further confirming that the expression of miR-381 and miR-495 is negatively correlated with MDR1 expression and function.

### A miR cluster on chromosome 14q32.31is down-regulated in K562/ADM cells

**Figure 5:**
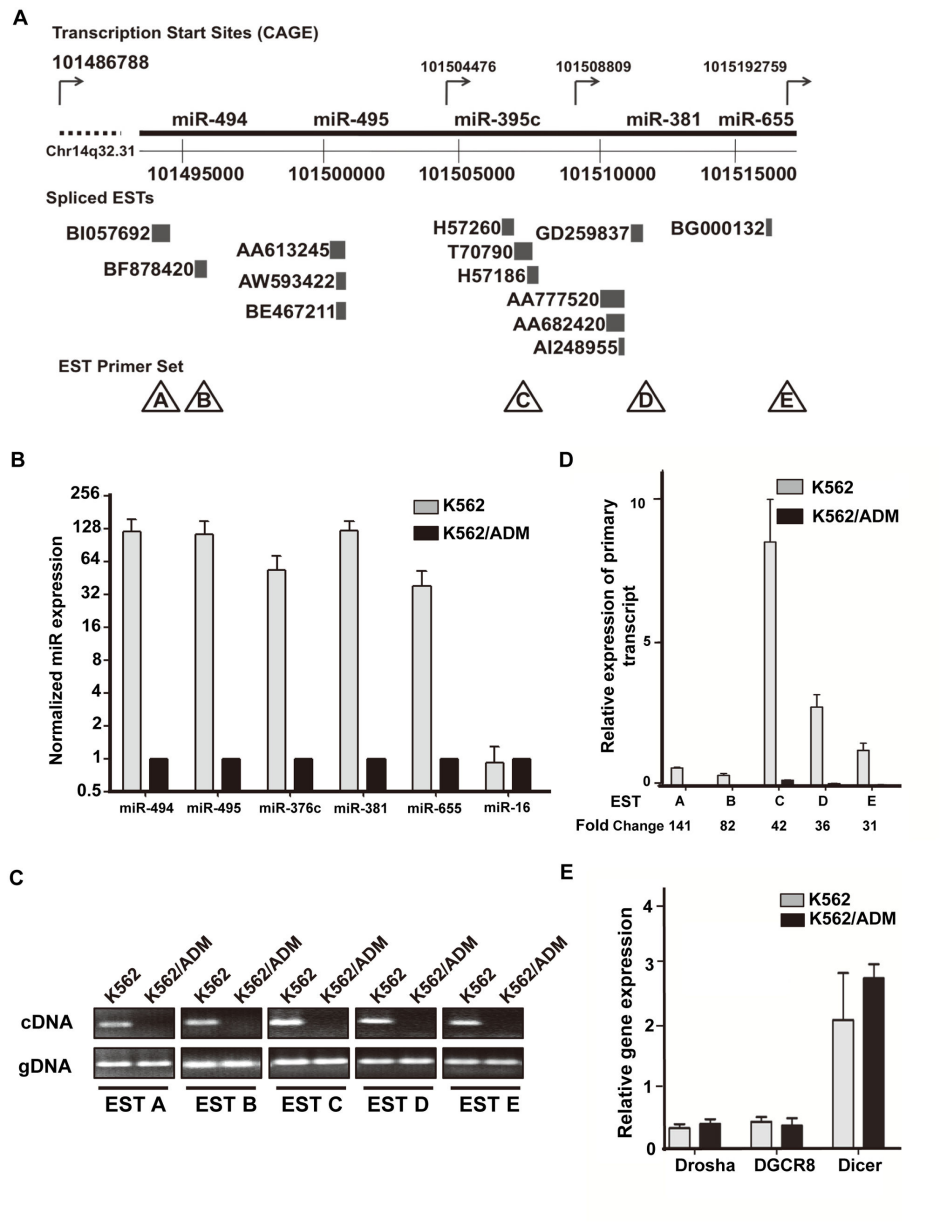
Expression of primary transcripts in the miR cluster on chromosome 14q32.31. (A) Schematic representation of known EST sites and selected miRs shown in UCSC genome browser (Human Feb. 2009 (GRCh37/hg19)) at chromosome region 14q32.31. EST primer sets A to E are indicated in triangles below each EST. Transcription start sites sourced from FANTOM CAGE tags are shown as arrows on the top of the 14q32.31 region. (B) Expression of selected miRs (miR-494, miR-495, miR-376c, miR-381 and miR-655) in K562 and K562/ADM cells was validated by stem-loop real-time PCR. The Ct values of these miRs were normalized to that of miR-425 whose level of expression was similar in the two cell lines. The relative expression of miR-494, miR-495, miR-376c, miR-381, miR-655 and miR-16 in K562/ADM cells was calculated. The results are shown as the mean ± SD of three independent experiments. (C) PCR products were resolved on a 2% agarose gel. (D) Expression of the primary transcripts in both K562 and K562/ADM was determined by RT-PCR using primers designed against ESTs A to E. The data were normalized to GAPDH. The results are shown as the mean ± SD of three independent experiments. Quantification of the change of expression level in K562/ADM cells relative to K562 cells is shown on the bottom. (E) Expression of three human RNA nuclease genes, Drosha, DGCR8 and Dicer, in both K562 and K562/ADM cells, was determined by real-time PCR. The expression values were normalized to GAPDH, and are shown as the mean ± SD of three independent experiments.

Both miR-381 and miR-495 were found to be located within a miR cluster spanning a distance of 20 kb in an imprinted region at chromosome location 14q32.31 based on the UCSC human genome assembly (GRCh37/hg19, February 2009, Figure 5A). All miRs within the cluster were down-regulated in K562/ADM cells according to our miRNAome profiling (Figure 5A and Table 2). Importantly, reduced expression of a number of other miRs in this region (including miR-494, miR-376c and miR-655 in addition to miR-381 and miR-495) was confirmed in K562/ADM cells by stem-loop real-time PCR analysis (Figures 3C and 5B).

Interestingly, approximately 13 ESTs were identified, from the UCSC hg19 human genome assembly, in the surrounding region of the miR-495/miR-381 cluster. Some miRs overlap with the ESTs; for example, miR-495 matches part of the sequence of EST AA613245 and miR-655 maps to EST BG000132 (Figure 5A). To measure the expression of the primary transcripts for miR-381 and miR-495, primer sets designated A to E were designed based on the known sequences of the ESTs (Figure 5A and Table S6). Reverse transcriptase PCR products on 2% agarose gels showed decreased EST expression in K562/ADM cells. However, analysis of genomic DNA with the same primer sets showed that this genomic region was not deleted in the K562/ADM cells (Figure 5C), indicating that the loss of expression occurred at the level of expression of the primary transcripts across this genomic region. The real-time PCR results presented in Figure 5D show the expression levels of these ESTs in K562 and K562/ADM cells with Ct values normalized to GAPDH. All the ESTs displayed a clear reduction in expression in the MDR cells with fold changes of 141, 82, 42, 36 and 31-times for A, B, C, D and E respectively.

As can be seen in Figure 5D, all these ESTs had relatively large fold decreases in MDR cells. The results also suggest that ESTs A and B may be transcribed from the same primary transcript that is different from that for EST C, while ESTs D and E might be transcribed from yet another primary transcript. This hypothesis is consistent with the transcription start sites sourced from FANTOM cap analysis gene expression (CAGE) tags [25] depicted in Figure 5D. ESTs A and B may share a common TSS starting at chr14:101486788 and encode the primary transcript for miR-494 and miR-495; EST C and miR-376c may share the same TSS (chr14:101504476) while ESTs D and E together with miR-381 and miR-655 may be transcribed from chr14:101508809. Therefore, we conclude that miR-381 and miR-495 may be the products of two different primary transcripts.

Drosha, DGCR8 and Dicer are known to regulate the processing of primary transcripts encoding miRs into mature miRs in a stepwise manner. Thus, examining the expression of Drosha, DGCR8 and Dicer in K562 and K562/ADM cells was an essential step to determine whether the down-regulation of miR-381 and miR-495 was related to dysregulation of these endonucleases. Real-time PCR with primers designed to uniquely target Drosha, DGCR8 and Dicer was conducted to determine their mRNA expression (Figure 5E). There is no evidence of differential expression of these endonucleases between K562 and K562/ADM cells, which suggests that down-regulation of miR-381 and miR-495 was not related to changes in the miR processing system. Since both the mature and the pre-miRs are down-regulated but their processing system appears to be normally expressed, it is possible that these results derive from decreased expression of the primary transcripts encoding these miRs in MDR cells.

## Discussion

A number of studies have revealed that miRs play critical roles in the acquisition of MDR by cancer cells [14,15,26]. In our study, we obtained a genome-wide profile of drug-induced miRs in human leukemia K562 cells using next-generation sequencing and identified and validated two miRs, miR-381 and miR-495, that may play a role in regulating the *MDR1* gene in leukemia cells. These two miRs were strongly down-regulated in two MDR K562 cell lines derived by treatment with ADM and VBL respectively, which suggests the possibility of diagnostic or prognostic applications as well as constituting potentially novel targets for therapy. *MDR1*/P-gp expression can be altered, either directly or indirectly, by modulating the levels of miR-381 or miR-495 in K562/ADM cells, hence affecting the substrate efflux function of P-gp, demonstrating the capacity of miR treatment to increase drug uptake in MDR leukemia cells.

Although our knowledge of the biology of miRs has advanced rapidly in recent years, much of the focus has been on their processing, targeting and regulatory mechanisms. Less is known about miR precursors, especially their primary transcripts. In this study, we have shown that both the mature forms and precursors of miR-381 and miR-495 are down-regulated in K562/ADM cells (Figure 3C). Because of the decreased expression of nearby ESTs (Figure 5), we draw the conclusion that the dysregulation of mature miRs appears to occur at the level of the primary transcripts as suggested by previous research [27–30]. Furthermore, the different expression patterns of the ESTs examined here and the CAGE TSSs sourced from FANTOM [25] suggest that miR-381 and miR-495 originate from different primary transcripts both of which are decreased in expression.

MiR-381 and miR-495 are both located in a cluster of miRs grouped within a 20 kb genomic region located at chromosome region 14q32.31. There is limited information available on miR clusters in general, although between 30-50% of miR genes are found to occur in clusters in zebrafish, mouse and human [31–33]. Clustered miRs are predicted to be transcribed as polycistrons and to have similar expression patterns [34,35]. A large cluster containing 40 miRs is located in the ^~^1 Mb imprinted 14q32 domain in humans as predicted by Seitz et al. [38] and the candidate miR cluster down-regulated in K562/ADM cells in this study falls into this region. Thus our data indicate that these miRs may be regulated by a common mechanism and warrants further investigation.

Many miR genes are located in genomic regions that are involved in chromosomal deletions or amplifications in human cancers [36,37]. We found that expression of ESTs and miRs in chromosomal region 14q32.31 is down-regulated in K562/ADM cells, suggesting the existence of a chromosomal deletion, insertion or translocation resulting from anticancer drug treatment. However, PCR using genomic DNA indicated that this region was intact in K562/ADM cells, excluding the possibility of a large chromosomal deletion. It has been reported that chromosome 14q32 is an unstable locus, with frequent translocations in cancer. For example, a t(14,18) (q32; q21) chromosomal translocation has been characterized in over 60% of human follicular lymphomas [38]; 28% of adult T-cell leukemia/lymphomas display a chromosome 14q32 translocation [39] while 20% of childhood T-cell acute lymphoblastic leukemias have a t(5;14) (q35; q32) chromosomal translocation [40]. Hence, we speculate that a translocation might be the cause of the dysregulated miR cluster in MDR leukemia cells although we do not have direct evidence of this and the details of a possible deletion, insertion or translocation in this region of chromosome 14 have yet to be investigated. It is thought that chromosomal instability is also related to the DNA methylation and histone modification that plays a critical role in epigenetic silencing of genes [41]. Therefore, we propose the alternative hypothesis that this region could be subject to epigenetic regulation in MDR leukemia cells leading to repression of miR expression.

Another point worth noting is that miR-381, miR-495 and their clusters are located 40 kb downstream of clusters of small nucleolar RNA (snoRNAs). This is another class of small non-coding RNA molecules that primarily guide chemical modifications of other RNAs [42]. In the human genome, at least two snoRNA clusters belonging to the C/D box class of snoRNAs are found in tandem repeats within the imprinted loci at chromosome 14q32 and chromosome 15q11q13 [43,44]. Chromosome 14q32 contains 9 copies of SNORD113 and 31 copies of SNORD114 within the introns of a tissue-specific non-coding human MEG8 RNA transcript. The functional roles of these snoRNAs have yet to be investigated, although it has been suggested that they play a role in the evolution and/or the mechanism of epigenetic imprinting [43]. Interestingly, these snoRNA and the miR clusters mapped to the human genome in the same orientation. Furthermore, 23 out of a total of 41 snoRNAs located down-stream of SNORD113 and SNORD114 at chromosome region 14q32 were found to be significantly down-regulated in K562/ADM cells (data not shown), in agreement with the pattern of down-regulation of the miR cluster. Whether down-regulation of the snoRNA and miR clusters in K562/ADM cells is driven by a common mechanism remains to be determined.

As a therapeutic strategy to overcome MDR, the use of miR-381 and miR-495 may have some advantages over conventional chemical inhibitors because they are native molecules in normal human cells. Also, they are small hydrophilic molecules, and thus able to modulate the expression of P-gp in aqueous environments in contrast to chemical inhibitors that are usually hydrophobic and function in the lipid phase[45]. Another possible advantage of a miR-based therapy would be the ability of a single miR to influence multiple cellular pathways by targeting the 3’UTRs of hundreds of different mRNAs [46,47]. However, the ability to affect a broad range of targets might also be a double-edged sword since any biological pathways regulated by these target genes would be affected, if and when the expression of miR-381 or miR-495 is changed, and might cause unwanted side effects. Therefore we employed RT-PCR to examine changes in gene expression as a result of down-regulation of miR-381 or miR-495. Several genes including ZEB1, TET1, ARID4B, MECOM, ZFPM2, that have putative miR binding sites and that may be targets of both miR-381 and miR-495 were selected based on the microRNA.org database. We found no obvious changes in the expression of these genes after transfection compared with the *MDR1* gene which, as expected, showed significantly decreased expression (Figure S3, note: log scale on y-axis). A possible explanation could be that miR-381 and miR-495 primarily target the *MDR1* gene with the other genes listed above being minor targets. However, we have only tested the expression of a limited set of genes in this study and it is possible that we have gained an incomplete view of the effects of modulating intracellular miR-381 or miR-495 levels on the expression of a broader range of genes. It would be interesting to widen the scope and search for other affected genes in a future study.

In conclusion, miR-381 and miR-495 are located within a cluster of dozens of down-regulated miRs on chromosome 14q32.21 and may be functionally important in the development of MDR in K562/ADM and K562/VBL cells. The naturally unstable nature of the chromosome 14q32 region may contribute to dysregulation of the primary transcripts of miR-381 and miR-495, and thus lead to decreased expression of the mature miR-381, miR-495 and the surrounding cluster of miRs. Therapeutic intervention with either miR-381 or miR-495 targeting P-gp expression may be possible, but more evidence would be needed to support this proposal. Use of a miR-based therapy, in addition to conventional chemotherapeutic agents, could potentially provide a next-generation approach to treatment, and provide many benefits for cancer patients, particularly for those with leukemia.

## Supporting Information

Figure S1 **Phenotypes of parental and MDR K562 cells.** (A) Morphological changes after treatment of K562 cells with ADM or VBL. (B) mRNA expression of MDR1 in both K562 and K562/VBL cells was determined by real time PCR. Expression values were normalized to GAPDH, and are shown as the mean ± SD of three independent experiments. (C) P-gp expression in both K562 and K562/VBL cells was determined by FACS analysis and is shown in the histogram.
(TIF)

Figure S2 **MDR1 3’-UTR target assay for miR-369-3p and miR-376a.** Relative GFP fluorescence intensity compared to internal control (DsRed) was analyzed by FACS after co-transfection with mimics of miR-369-3p, miR-376a or negative control. Both miR-369-3p and miR-376a show strongly reduced expression in K562/ADM cells and are not predicted to target the MDR1 gene.
(TIF)

Figure S3 **Expression of genes targeted by miR-381 and/or miR-495.** Expression of representative genes targeted by both miR-381 and miR-495 in K562 cells and K562/ADM cells with or without transfection with miR mimics was determined by real time-PCR. Expression values were normalized to GAPDH, and are shown as the mean ± SD of three independent experiments.
(TIF)

Table S1 **Primers to validate gene expression by real time PCR.**
(DOC)

Table S2 **Primers to validate mature miRs by stem-loop real-time PCR.**
(DOC)

Table S3 **Sequences of miR mimics.**
(DOC)

Table S4 **Sequences of miR inhibitors.**
(DOC)

Table S5 **Up-regulated miRs with >10-fold increase in expression in K562/ADM cells.**
(DOC)

Table S6 **Primers to validate EST expression by real time PCR.**
(DOC)
