# Supplementary figures and images for: Changes in the Expression of miR-381 and miR-495 Are Inversely Associated with the Expression of the MDR1 Gene and Development of Multi-Drug Resistance
Source: PLoS One. 2013 Nov 26;8(11):e82062. doi: 10.1371/journal.pone.0082062 (PMC3841137; doi:10.1371/journal.pone.0082062)

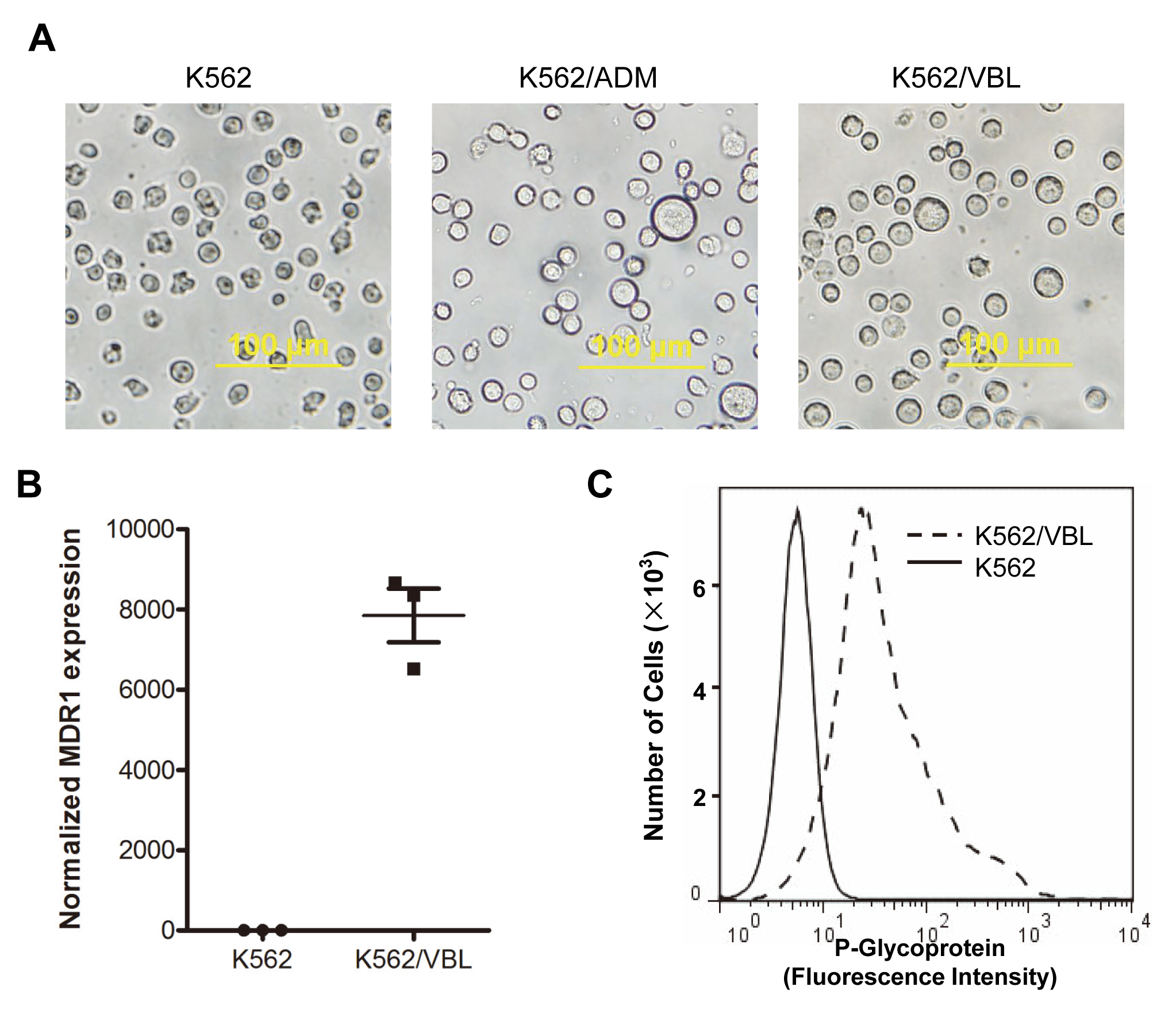

Supplement: Figure S1 — Phenotypes of parental and MDR K562 cells. (A) Morphological changes after treatment of K562 cells with ADM or VBL. (B) mRNA expression of MDR1 in both K562 and K562/VBL cells was determined by real time PCR. Expression values were normalized to GAPDH, and are shown as the mean ± SD of three independent experiments. (C) P-gp expression in both K562 and K562/VBL cells was determined by FACS analysis and is shown in the histogram. (TIF) [file pone.0082062.s001.tif]

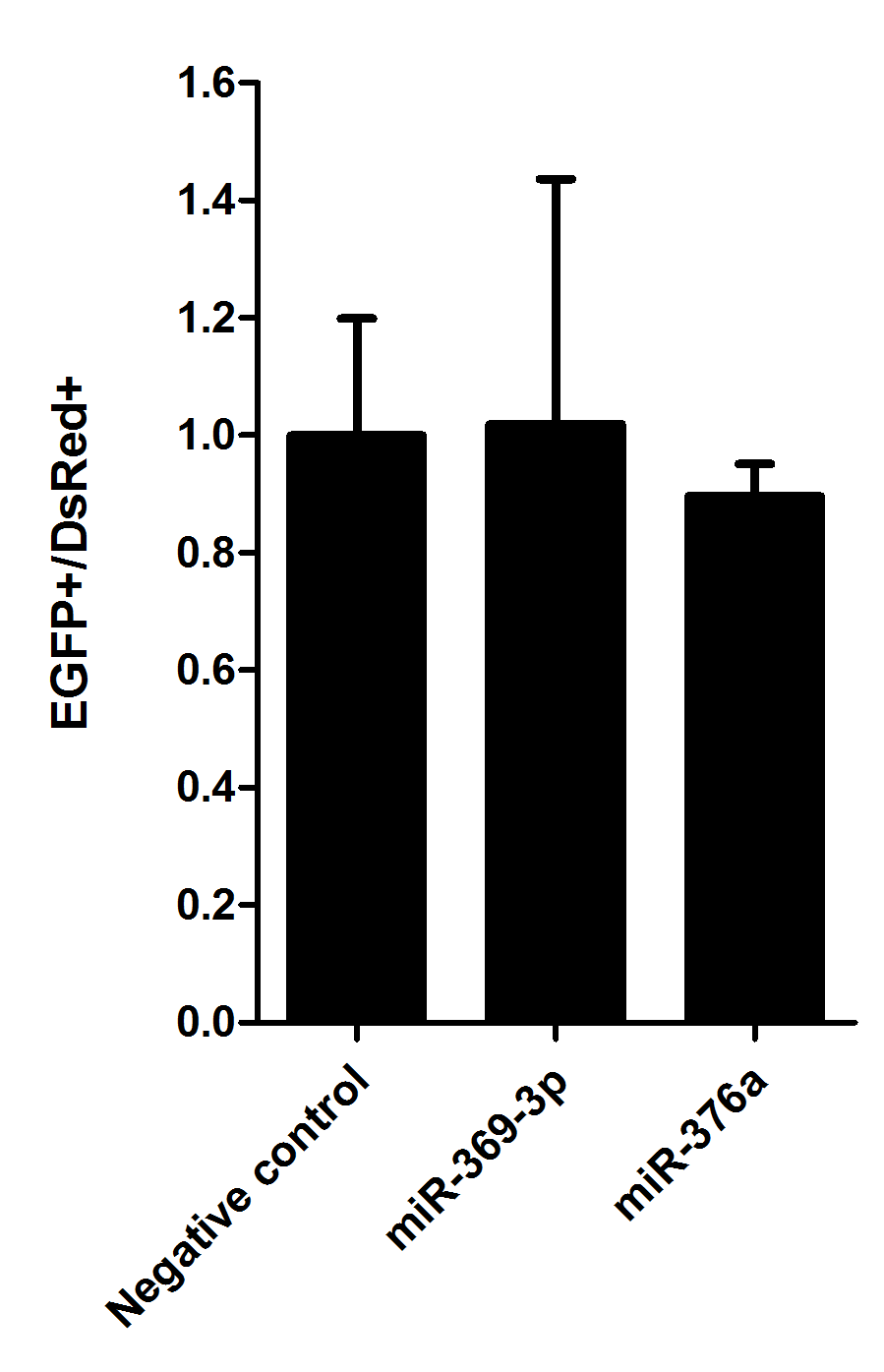

Supplement: Figure S2 — MDR1 3’-UTR target assay for miR-369-3p and miR-376a. Relative GFP fluorescence intensity compared to internal control (DsRed) was analyzed by FACS after co-transfection with mimics of miR-369-3p, miR-376a or negative control. Both miR-369-3p and miR-376a show strongly reduced expression in K562/ADM cells and are not predicted to target the MDR1 gene. (TIF) [file pone.0082062.s002.tif]

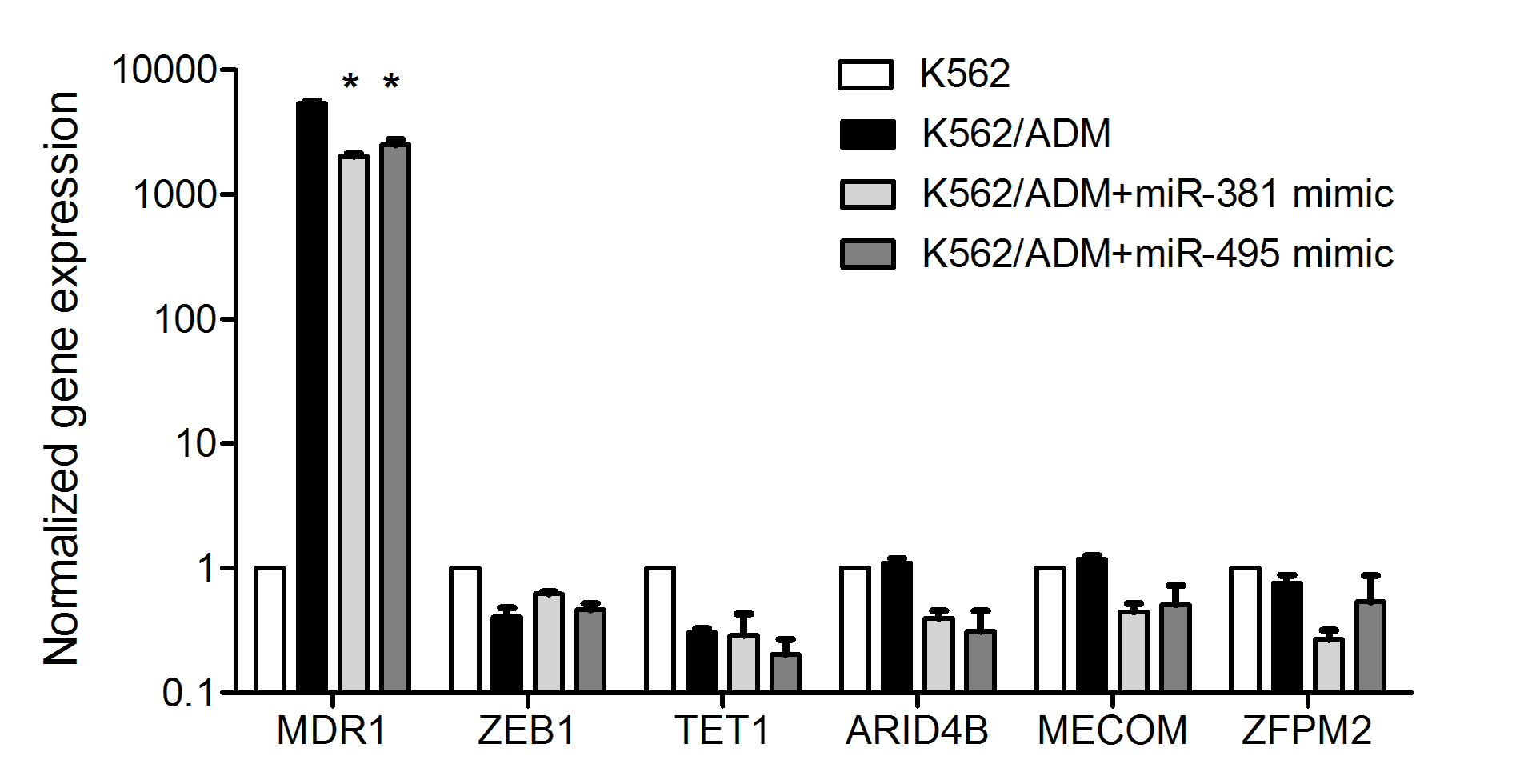

Supplement: Figure S3 — Expression of genes targeted by miR-381 and/or miR-495. Expression of representative genes targeted by both miR-381 and miR-495 in K562 cells and K562/ADM cells with or without transfection with miR mimics was determined by real time-PCR. Expression values were normalized to GAPDH, and are shown as the mean ± SD of three independent experiments. (TIF) [file pone.0082062.s003.tif]
