# Supplementary material for: Changes in the Expression of miR-381 and miR-495 Are Inversely Associated with the Expression of the MDR1 Gene and Development of Multi-Drug Resistance
Source: PLoS One. 2013 Nov 26;8(11):e82062. doi: 10.1371/journal.pone.0082062 (PMC3841137; doi:10.1371/journal.pone.0082062)
Supplement: Table S1 — Primers to validate gene expression by real time PCR. (DOC) [file pone.0082062.s004.doc]

Table S1. Primers to validate gene expression by real time PCR.

| **Primers** | **Sequences (5’→3’)** |
| --- | --- |
| GAPDH For | TGGTATGACAACGAATTTGG |
| GAPDH Rev | TCTACATGGCAACTGTGAGG |
| MDR1 For | CCCATCATTGCAATAGCAGG |
| MDR1 Rev | GTTCAAACTTCTGCTCCTGA |
| Drosha For | CATGCACCAGATTCTCCTGTA |
| Drosha Rev | GTCTCCTGCATAACTCAACTG |
| DGCR8 For | TATCAGATCCTCCACGAGTG |
| DGCR8 Rev | TCTTGGAGCTTGCTGAGGAT |
| Dicer For | GTACGACTACCACAAGTACTTC |
| Dicer Rev | ATAGTACACCTGCCAGACTGT |
| ZEB1 For | CAGGTTGCTCCTTCTTCCTG |
| ZEB1 Rev | TGCAGGAGCTGAGAGTCAGA |
| Tet1 For | CACACCAGCTCCACTGAAGA |
| Tet1 Rev | TCCATCACAGGAGCAGACAG |
| ARID4B For | ACTGCACAGTGGCACAAAAA |
| ARID4B Rev | CCAGACACACAATTCCCAGA |
| ZFPM2 For | TGCTGGACTATCACGAGTGC |
| ZFPM2 Rev | GGTTGCTAGGTGGGATGAAA |
| CD9 For | TCAGCTTACATCCCTGAGCA |
| CD9 Rev | AAACCAAAGCAAACCAAACC |
