# Supplementary material for: Changes in the Expression of miR-381 and miR-495 Are Inversely Associated with the Expression of the MDR1 Gene and Development of Multi-Drug Resistance
Source: PLoS One. 2013 Nov 26;8(11):e82062. doi: 10.1371/journal.pone.0082062 (PMC3841137; doi:10.1371/journal.pone.0082062)
Supplement: Table S2 — Primers to validate mature miRs by stem-loop real-time PCR. (DOC) [file pone.0082062.s005.doc]

Table S2. Primers to validate mature miRs by stem-loop real-time PCR.

| **Primers** | **Sequences (5’→3’)** |
| --- | --- |
| SL-miR-494 | GTCGTATCCAGTGCAGGGTCCGAGGTATTCGCACTGGATACGACgaggtt |
| SL-miR-495 | GTCGTATCCAGTGCAGGGTCCGAGGTATTCGCACTGGATACGACaagaag |
| SL-miR-376c | GTCGTATCCAGTGCAGGGTCCGAGGTATTCGCACTGGATACGACacgtgg |
| SL-miR-381 | GTCGTATCCAGTGCAGGGTCCGAGGTATTCGCACTGGATACGACacagag |
| SL-miR-655 | GTCGTATCCAGTGCAGGGTCCGAGGTATTCGCACTGGATACGACAAAGAG |
| SL-miR-425 | GTCGTATCCAGTGCAGGGTCCGAGGTATTCGCACTGGATACGACtcaacg |
| SL-miR-16 | GTCGTATCCAGTGCAGGGTCCGAGGTATTCGCACTGGATACGACcgccaa |
| SL-miR-494 For | ggggTGAAACATACACGGGA |
| SL-miR-495 For | ggggAAACAAACATGGTGCA |
| SL-miR-376c For | ggggAACATAGAGGAAATTC |
| SL-miR-381 For | ggggTATACAAGGGCAAGCT |
| SL-miR-655 For | GGGGATAATACATGGTTAAC |
| SL-miR-425 For | ggggAATGACACGATCACTCC |
| SL-miR-16 For | ggggTAGCAGCACGTAAATA |
| Universal Rev | GTGCAGGGTCCGAGGT |
