# Supplementary material for: Changes in the Expression of miR-381 and miR-495 Are Inversely Associated with the Expression of the MDR1 Gene and Development of Multi-Drug Resistance
Source: PLoS One. 2013 Nov 26;8(11):e82062. doi: 10.1371/journal.pone.0082062 (PMC3841137; doi:10.1371/journal.pone.0082062)
Supplement: Table S3 — Sequences of miR mimics. (DOC) [file pone.0082062.s006.doc]

Table S3. Sequences of miR mimics.

| **miR ID** | **Sequences** | |
| --- | --- | --- |
|  | **Sense (5’→3’)** | **Antisense (5’→3’)** |
| hsa-miR-381 mimics | UAUACAAGGGCAAGCUCUCUGU | AGAGAGCUUGCCCUUGUAUAUU |
| hsa-miR-495 mimics | AAACAAACAUGGUGCACUUCUU | GAAGUGCACCAUGUUUGUUUUU |
| hsa-miR-369-3p mimics | AAUAAUACAUGGUUGAUCUUU | AGAUCAACCAUGUAUUAUUUU |
| hsa-miR-376a mimics | AUCAUAGAGGAAAAUCCACGU | GUGGAUUUUCCUCUAUGAUUU |
| Negative control | UUCUCCGAACGUGUCACGUTT | ACGUGACACGUUCGGAGAATT |
