# Supplementary material for: Changes in the Expression of miR-381 and miR-495 Are Inversely Associated with the Expression of the MDR1 Gene and Development of Multi-Drug Resistance
Source: PLoS One. 2013 Nov 26;8(11):e82062. doi: 10.1371/journal.pone.0082062 (PMC3841137; doi:10.1371/journal.pone.0082062)
Supplement: Table S4 — Sequences of miR inhibitors. (DOC) [file pone.0082062.s007.doc]

Table S4. Sequences of miR inhibitors.

| **Mir ID** | **Sequences (5’→3’)** |
| --- | --- |
| hsa-miR-381 inhibitor | ACAGAGAGCUUGCCCUUGUAUA |
| hsa-miR-495 inhibitor | AAGAAGUGCACCAUGUUUGUUU |
| Negative control | CAGUACUUUUGUGUAGUACAA |
