# Supplementary material for: Changes in the Expression of miR-381 and miR-495 Are Inversely Associated with the Expression of the MDR1 Gene and Development of Multi-Drug Resistance
Source: PLoS One. 2013 Nov 26;8(11):e82062. doi: 10.1371/journal.pone.0082062 (PMC3841137; doi:10.1371/journal.pone.0082062)
Supplement: Table S5 — Up-regulated miRs with >10-fold increase in expression in K562/ADM cells. (DOC) [file pone.0082062.s008.doc]

Table S5. Up-regulated miRs with >10-fold increase in expression in K562/ADM cells.

| **miR ID** | **Normalized reads** | | **P value** | **Fold change (up-regulation)** |
| --- | --- | --- | --- | --- |
|  | **K562** | **K562/ADM** |  |  |
| hsa-miR-584 | 6.325574148 | 687.2103462 | 0.00183258 | 109 |
| hsa-miR-514 | 0.90365345 | 75.25008622 | 0.00444475 | 83 |
| hsa-miR-3681* | 1.807306899 | 56.43756466 | 0.01775992 | 31 |
| hsa-miR-488 | 0.90365345 | 27.66547287 | 0.02484657 | 31 |
| hsa-miR-1269 | 0.90365345 | 25.45223504 | 0.02840621 | 28 |
| hsa-miR-3117 | 29.82056384 | 663.971349 | 0.01764223 | 22 |
| hsa-miR-375 | 177.1160761 | 3887.552248 | 0.01067047 | 22 |
| hsa-miR-202* | 3.614613799 | 78.56994296 | 0.02307016 | 22 |
| hsa-miR-508-3p | 1.807306899 | 37.62504311 | 0.03528011 | 21 |
| hsa-miR-3681 | 328.9298557 | 6119.6026 | 0.01061964 | 19 |
| hsa-mir-383* | 0.90365345 | 13.27942698 | 0.07402589 | 15 |
| hsa-miR-184 | 226.8170159 | 2940.286457 | 0.02985391 | 13 |
| hsa-miR-135b | 150.9101261 | 1846.946969 | 0.03823105 | 12 |
| hsa-miR-149 | 9.036534497 | 105.1287969 | 0.04506416 | 12 |
| hsa-miR-509-3p | 1.807306899 | 19.91914047 | 0.09348999 | 11 |
| hsa-miR-489 | 3.614613799 | 37.62504311 | 0.07426698 | 10 |
| hsa-miR-338-3p | 1.807306899 | 18.81252155 | 0.10127334 | 10 |
| hsa-miR-146a | 215.069521 | 2181.145881 | 0.04864756 | 10 |
