# Supplementary material for: Changes in the Expression of miR-381 and miR-495 Are Inversely Associated with the Expression of the MDR1 Gene and Development of Multi-Drug Resistance
Source: PLoS One. 2013 Nov 26;8(11):e82062. doi: 10.1371/journal.pone.0082062 (PMC3841137; doi:10.1371/journal.pone.0082062)
Supplement: Table S6 — Primers to validate EST expression by real time PCR. (DOC) [file pone.0082062.s009.doc]

Table S6. Primers to validate EST expression by real time PCR.

| **Primers** | **Sequences (5’→3’)** |
| --- | --- |
| EST-A For | ATGCCCTGGACAGATAGTGC |
| EST-A Rev | TGGCATGACATCAAGGATTC |
| EST-B For | AGAAGATGTGGGAGGACGTG |
| EST-B Rev | GGCAGGATTTGAAACCAAA |
| EST-C For | GTGTGGATGGTTGACCACAG |
| EST-C Rev | GGTAGGCCCTTTTTGCTAGG |
| EST-D For | GTGTCAACGGCTCTGTGAGA |
| EST-D Rev | CACCATCTCCTTCCCAGTGT |
| EST-E For | AGAGGCCATGGTTTTCGTTT |
| EST-E Rev | CGAGGCAGAGTCTGATATTCAA |
